# Differences in evolutionary pressure acting within highly conserved ortholog groups

**DOI:** 10.1186/1471-2148-8-208

**Published:** 2008-07-17

**Authors:** Teresa M Przytycka, Raja Jothi, L Aravind, David J Lipman

**Affiliations:** 1 National Center for Biotechnology Information, National Library of Medicine, National Institutes of Health, Bethesda, MD 20894, USA; 2 National Heart, Lung, and Blood Institute, National Institutes of Health, Bethesda, MD 20894, USA

## Abstract

**Background:**

In highly conserved widely distributed ortholog groups, the main evolutionary force is assumed to be purifying selection that enforces sequence conservation, with most divergence occurring by accumulation of neutral substitutions. Using a set of ortholog groups from prokaryotes, with a single representative in each studied organism, we asked the question if this evolutionary pressure is acting similarly on different subgroups of orthologs defined as major lineages (e.g. Proteobacteria or Firmicutes).

**Results:**

Using correlations in entropy measures as a proxy for evolutionary pressure, we observed two distinct behaviors within our ortholog collection. The first subset of ortholog groups, called here informational, consisted mostly of proteins associated with information processing (i.e. translation, transcription, DNA replication) and the second, the non-informational ortholog groups, mostly comprised of proteins involved in metabolic pathways. The evolutionary pressure acting on non-informational proteins is more uniform relative to their informational counterparts. The non-informational proteins show higher level of correlation between entropy profiles and more uniformity across subgroups.

**Conclusion:**

The low correlation of entropy profiles in the informational ortholog groups suggest that the evolutionary pressure acting on the informational ortholog groups is not uniform across different clades considered this study. This might suggest "fine-tuning" of informational proteins in each lineage leading to lineage-specific differences in selection. This, in turn, could make these proteins less exchangeable between lineages. In contrast, the uniformity of the selective pressure acting on the non-informational groups might allow the exchange of the genetic material via lateral gene transfer.

## 1. Background

Previous studies have shown that proteins are under purifying selection which enforces a certain stasis in terms of sequence and function. Much less frequently they are subject to episodes of positive selection, which are typified by accelerated sequence divergence and corresponding functional shifts [1-6]. A basic assumption in molecular evolution is that the selective pressure represents functional constraints and is correlated with evolutionary conservation [2]. Direct measurement of the functional constraints is not straight-forward; however its effects may be estimated through sequence conservation. For closely related species, selective pressure is usually measured using a nucleotide alignment and the ratio of non-synonymous over synonymous (silent) substitutions [7,8]. For more divergent species, the purifying selective pressure can be measured through the imprint it makes on the multiple sequence alignment of proteins in an ortholog group.

We consider a set of ortholog groups, which are conserved over a broad spectrum of prokaryotes. We additionally require that selected proteins do not have paralogs in studied organisms. Hence a change in biological function of the proteins within each group is unlikely. Within such an ortholog group, the general expectation is that the main evolutionary force is purifying selection, which is reflected as sequence conservation, with most divergence between the orthologs arising from neutral substitutions. Sequences in a given ortholog group can be further divided into subgroups each comprised of different monophyletic lineages, for example Proteobacteria, Firmicutes, and Archaea. We were interested in understanding whether the selective pressure was similar across the different subgroups, and if the measure of selective pressure acting on one subgroup is predictive of that acting on another within the same ortholog group. We present results of this study and provide evidence that Lateral Gene Transfer (LGT) might have a noticeable, apparently non-intuitive, effect on such extrapolations of selective pressure. We outline below the basic approach used in this study.

### Approach

Selective pressure, defined as the influence of natural selection in enforcing conservation or in favoring divergence in protein or DNA sequence, has been a observed to be the basis for conservation patterns across different subfamilies in a family of homologous molecules [9]. It has been measured previously using the entropy of individual positions in multiple sequence alignment as a proxy [9-11].

The ortholog groups used in this study satisfies the uniqueness condition, i.e. none of the proteins has a confounding paralog within the same genome, which cannot be differentiated from the true ortholog. Therefore, it is prudent to assume that proteins from such ortholog group perform a comparable function in the corresponding organisms. Consequently, significant deviations from the uniformity in the pattern of sequence conservation in different subgroups can be attributed to differences in effects of selection within these subgroups. We stress that observed differences in the sequence conservation patterns do not directly provide information on the causes for the inferred differences (for example in terms of variation in Ks/Kn ratio) nor the reasons for which the constraints might be different in different clades. Possible reasons may vary from differences in the environment to lineage specific "fine tuning" of proteins functioning as parts of multi-protein complexes. To emphasize the connection to evolution on longer distances selective constraints measured in this way are also referred to as evolutionary pressure [10,11].

We represent variability in sequence conservation by an entropy profile – a vector constructed from multiple sequence alignment where the value at the *i*^th ^position in the vector equals the entropy of the corresponding column in the alignment. Thus the entries of the entropy profile vary with the sequence conservation. Consequently, given two subfamilies, it can be tested if their entropy profiles are correlated. Such correlation would be expected if both subfamilies ware subjected to the same evolutionary pressure.

It is important to keep in mind that entropy profile is shaped not only by selective constraints but also by evolutionary distances between the species. We control for this dependency by keeping the set of species fixed for all ortholog groups under study.

An ultimate test for uniformity of evolutionary constraints would require that one can predict the evolutionary pressure imposed on sequences in one subgroup *X *of an ortholog group based on information on a different subgroup, *N *(kNown), of this group. In this work, rather than predicting the entropy profile of *X *from the entropy profile of *N *we focus on the more modest task of predicting the Pearson's correlation coefficient, *cc*(*N*, *X*) between them. The value of the Pearson's correlation coefficient of entropy profiles of two subgroups of the same ortholog group provides a first estimation of the uniformity of the selective pressure between the groups. In contrast to evolutionary distance measures, correlation coefficient depends not only on the amount of evolutionary changes but also on the location of these changes in protein sequence.

**Figure 1:**
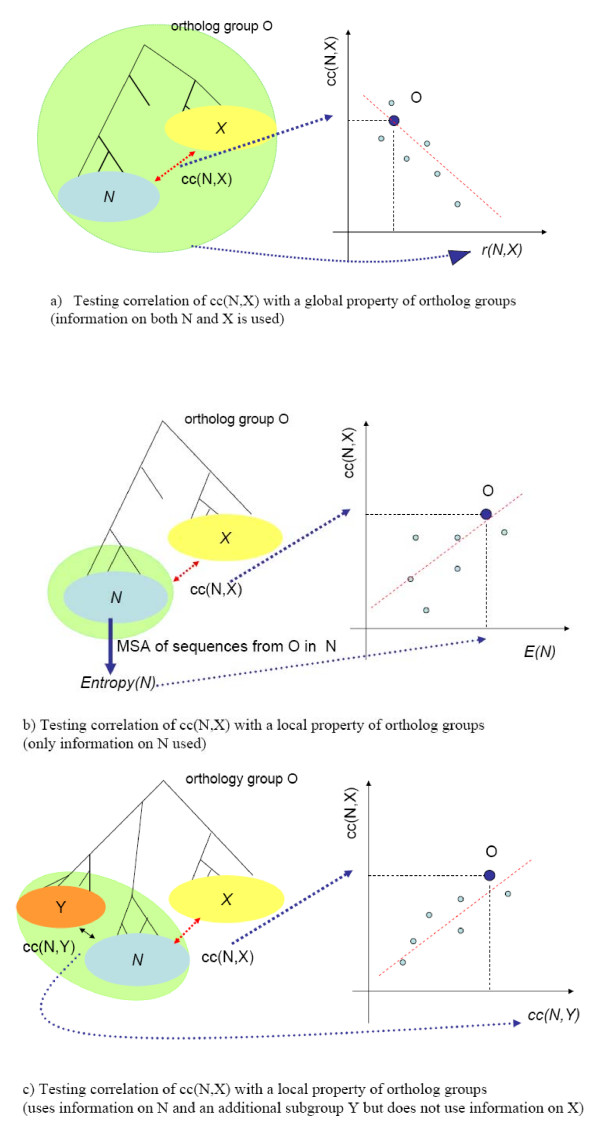
**The dependence of the Pearson correlation coefficient of entropy profiles cc(N, X) between two subgroups N and X of the same ortholog group and three different measurements of properties of ortholog group.** The green oval in each figure indicates the information used in the corresponding measurement a) global property: the ratio of distance between the last common ancestors of the genes in subgroups *X *and *N *and the average distance between sequences from the different subgroups. b) local property: the average entropy of the alignment of sequences in N c) the semi-local property: the correlation of cc(N, Y) with value of cc(N, Y) computed based on entropy profile of N to subgroup Y different than X.

We cast the question of uniformity of the evolutionary pressure within an ortholog group as a general question of predictability of the value of the correlation coefficient between entropy profiles of two its subgroups. More precisely, we ask if the Pearson's correlation coefficient, *cc*(*N*, *X*), between entropy profiles of two subgroups *N *and *X *can be expressed as a function of some measurable property (or properties) of ortholog subgroup *N*. We selected three such measurable properties: one "global", one "local" and one "semi-local" (Figure 1). For each of these three measurements we tested if the value of *cc*(*N*, *X*), is correlated (positively or negatively) with the given measurement. The first measurement (Figure 1a) is a value that approximates the ratio of the distance between the last common ancestors of the genes in subgroups *X *and *N *to the average distance between sequences from different subgroups. Thus this measurement uses information about both subgroups *X *and *N *and therefore we classified it as a global property. The second measurement estimates the level of conservation of sequences in *N *(Figure 1b). We defined this measurement as a local measurement, as it does not require any knowledge of the ortholog group beyond the properties of the sequences in the subgroup *N*. We estimated the conservation within ortholog subgroup, X, using two measures: average entropy and percent of perfectly conserved columns. Finally, the last, semi-local, measurement is based on value *cc*(*N*, *Y*) computed for *N *and another subgroup *Y *different from *X *(Figure 1c). So while it uses information about sequences other than these in the subgroup *N *(thus it is not local), however it doesn't use sequences in the subgroup *X*.

To delineate the relation between lateral gene transfer and universality of selective constraints, we identified the subset of ortholog groups with putative lateral gene transfers between different clades and examined specific properties of this set. Additionally, we performed a series of *in-silico *lateral gene transfers (s-LGTs). In these experiments a random member of a given subgroup is replaced with a random member from another subgroup of the same ortholog group. Then we measured the effect of such s-LGT on the correlation coefficient.

## 2. Results and discussion

This study utilizes three prokaryotic clades: Archaea, Proteobacteria, and Gram positive bacteria. In this paper we use A, P, and G to denote the subgroup of an ortholgy group restricted to the given clade (Archaea, Proteobacteria, and Gram positive bacteria respectively). The ortholog groups were extracted based on the COG database [12] and were filtered so that that each ortholog group has a unique homolog in each of the selected organisms. Such stringent restriction leads to the trade-off between the number of species in a clade and the number of ortholog groups in the study. After confirming high correlation between values of *cc(N, X) *for four and six species (R^2 ^was 86, 78, 80 depending on *N *and *X*, [see Additional file 1]) we concluded using the four-element clades should still provide reliable result and at the same time allow for considering a broader range of ortholog groups (see Methods). The set of 63 ortholog groups obtained in this way was divided further into the "informational groups" containing 37 ortholog groups associated with functions related to information processing (i.e. translation, ribosomal proteins, transcription, DNA replication) and the "non-informational groups" containing 26 remaining ortholog groups, which are mostly proteins involved in metabolism (see Additional file 2 for full description).

### 2.1. Negative correlation of *cc*(*N*, *X*) and the relative root distance – global measurement

**Figure 2:**
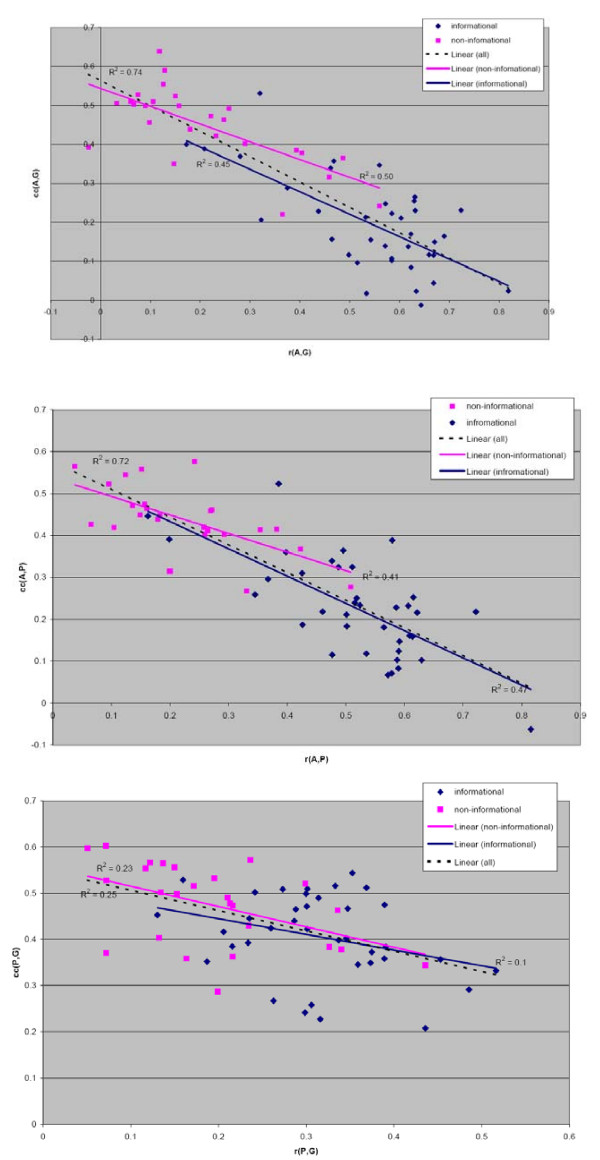
**The dependency of correlation between entropy profiles on the relative root distance for all three pairs of clades.** Informational groups are shown as navy diamonds and to the non-informational groups as magenta squares. The linear regression line for full set of points is shown with broken line.

First, we tested if *cc*(*N*, *X*) can be predicted from the information encoded in the phylogenic tree of the ortholog group. Specifically, we used a value referred to as the relative root distance *r*(*N*, *X*), approximating the ratio of the distance between last common ancestors of the two subgroups to the average evolutionary distances of the sequences in the two subgroups (see Methods for definition). Note, that by relying on the phylogenetic tree, this test uses information about both subgroups *N *and *X*. Thus this constitutes a global measurement of the ortholog groups (Figure 1a). We observed a negative correlation between *cc*(*N*, *X*) and the relative root distance *r*(*N*, *X*) (Figure 2). The coefficient of determination, R^2^, when one of the two subgroups belonged to Archaea ((*G*, *A*) and (*P*, *A*)) was respectively 0.74 and 0.72, while that where both subgroups corresponded to bacterial clades was 0.25. Hence, the value of *r*(*N*, *X*) is negatively correlated with *cc*(*N*, *X*) and thus can be used to predict the latter value. However, since *r*(*N*, *X*) is a global measure that uses information on both N and X we cannot conclude that *cc*(*N*, *X*) can be predicted from N alone.

This experiment suggested a strong dependency of the similarity of entropy profiles on the shape of the ortholog tree. In addition, it pointed out the first of a series of differences in the properties of the informational and non-informational groups: the relative root distances in the set of informational groups are on average larger than the relative root distances in the set of non-informational groups (see summary in Table 1).

**Table 1:** Average correlation between entropy profiles between various clades and average values of the entropy. P-values are computed based on the t-test

Correlation of entropy profiles			
	cc(G, A)	cc(P, A)	cc(P, G)

Average cc non-informational	0.45	0.42	0.47
Average cc informational	0.19	0.22	0.39
p-value for the difference	< 0.001	< 0.001	< 0.001

Relative root distance			

Average r non-informational	0.20	0.21	0.18
Average r informational	0.54	0.52	0.31
p-value for the difference	< 0.001	< 0.001	< 0.001

Entropy			

	Proteobacteria (P)	Gram Positive (G)	Archaea (A)

Average Entropy non-informational	0.65	0.65	0.76
Average Entropy informational	0.54	0.46	0.69
p-value for the difference	> 0.1	> 0.1	> 0.1

### 2.2. Dependency of *cc*(*N*, *X*) on sequence conservation in group N- local measurement

The previous test demonstrated a negative correlation between the relative root distance *r*(*N*, *X*) computed on the basis of pairwise distances between protein sequences in *X *and *N *and *cc*(*N*, *X*). Next, we tested if *cc*(*N*, *X*) is correlated with sequence divergence within the ortholog subgroup *N *(Figure 1b). For this purpose, we measured the correlation between negated average entropy *E*(*N*) of the subgroup *N *and the value of *cc*(*N*, *X*), for all choices of N and X (six experiments). We performed the same set of experiment using the percentage of perfectly conserved columns in *N, PC*(*N*), instead of E(N). We found that the two measures are strongly correlated (R^2 ^> 0.95 for all subgroups) and the results obtained using with either of the two measures were very consistent. Therefore, we focused on the relation between *E*(*N*) and *cc*(*N*, *X*). Out of the six experiments only pairs *E*(*P*), *cc*(*A*, *P*)) and (*E*(*G*), *cc*(*A*, *G*)) were correlated with R^2 ^> 0.1 (0.17 and 0.38 respectively).

Subsequently, we focused on comparing average properties of informational and non-informational groups. Although, on average, the entropy of non-informational subgroups is higher than that of informational subgroups (and the percent conservation lower) the difference is not statistically significant. In contrast, the values of *cc*(*N*, *X*) are significantly higher for non-informational groups (Table 1). This clear difference between the two ortholog groups is suggestive non-uniformity of constraints on the informational groups. These constraints might preserve certain mutations specific to particular subgroups within the informational ortholog groups. Another striking observation was that for non-informational ortholog groups, the average correlation coefficient is approximately the same for all pairs of clades suggesting an additional level of uniformity of the these groups.

### 2.3. Uncovering the relation between the *cc*(*N*, *X*) for different pairs of subgroups – semi-local measurement

**Figure 3:**
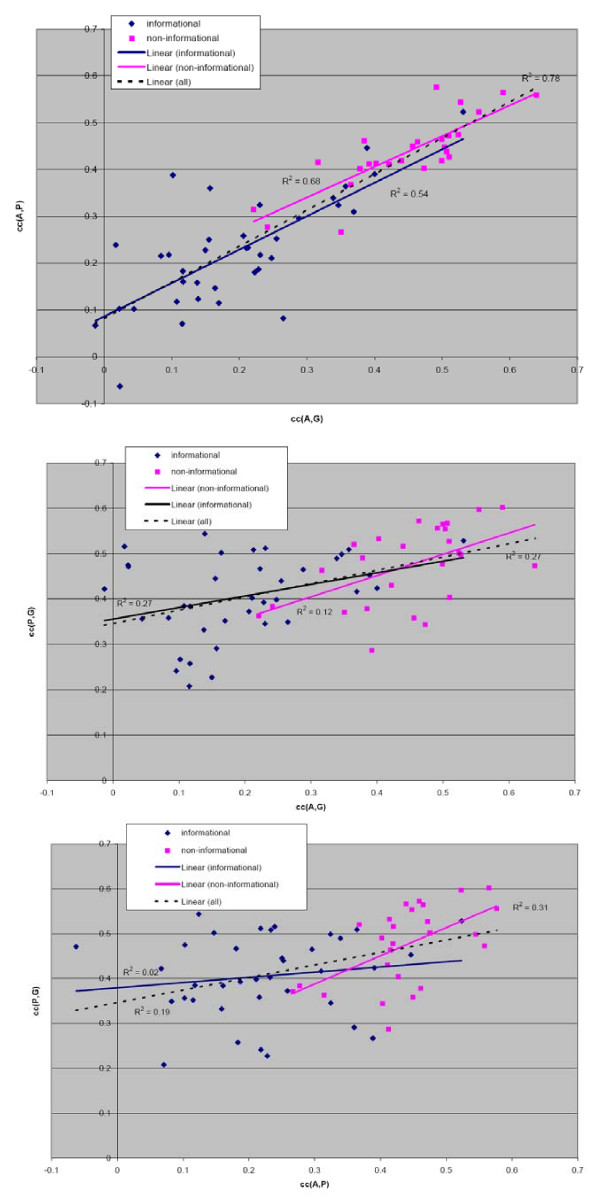
**The dependency between correlation profiles *cc(N, X) *and *cc(N, Y) *all three pairs of clades.** Informational groups are shown as navy diamonds and to the non-informational groups as magenta squares. The linear regression line for full set of points is shown with broken line.

Given the above observations, we sought to understand the separation of informational and non-informational groups in greater detail. We observed a reasonable correlation of our global measurement, relative root distance *r*(*N*, *X*), and *cc*(*N*, *X*) (Section 2.1). In contrast, the correlation between our local measurement, average entropy, E(N) and *cc*(*N*, *X*) was very low (Section 2.2). Therefore we considered an intermediate, semi-local, measurement of ortholog groups (Figure 1c). Specifically, we studied the dependency of the correlation *cc*(*N*, *X*) between and *cc*(*N*, *Y*) where *N, X*, and *Y *are different subgroups of the same ortholog group corresponding to distinct clades. The coefficients of determination, R^2^, for the correlation between *cc*(*N*, *Y*) and *cc*(*N*, *X*) for the three possible combinations of subgroups were 0.78, 0.27 and 0.19 depending on the subgroups, with the highest correlation for the pair (*cc*(*A*, *G*), *cc*(*A*, *P*)) and the lowest for the pair (*cc*(*P*, *G*), *cc*(*P*, *A*)) (Figure 3). Just as in the previous measurements, we found that informational and non-informational proteins have a distinct behavior with respect to this measure – the values for non-informational groups showed higher correlation. Specifically, the R^2 ^values for non-informational groups are 0.68, 0.27 and 0.31 (listed in the same order as above) while the corresponding values for the informational groups are 0.54, 0.11 and 0.02.

This provides yet more evidence for the observation that evolutionary pressure acts more uniformly on the non-informational groups than on the informational groups. These results also give further support to the observation that a significant fraction of the informational ortholog groups might be a subject to lineage specific evolutionary pressure. If so, this would imply that proteins in this group are not easily exchangeable between species through LGT. In contrast, the selective pressure acting on non-informational proteins is much more uniform and may more easily permit exchange of corresponding orthologs and corresponding xenologous displacement [13].

### 2.4. Lateral gene transfer and evolutionary pressure

The above observations suggested that proteins in informational ortholog groups may be less prone to exchange between lineages, while exchanges in the non-informational groups are more likely. To test if this indeed is the case, we constructed evolutionary trees for all ortholog groups and manually looked for deviations from the species tree, which would imply lateral gene transfer (LGT) between the clades (see Material and methods). We found that only 3 out of 37 informational group trees had a signature of such putative LGTs while most (18 out of 27) non-informational groups show such a signature of possible lateral gene transfer consistent with our expectation. We found that non-informational groups have higher correlation between *cc*(*N*, *X*) and *cc*(*N*, *Y*) than informational groups (Table 2). Surprisingly we observed lack of increased correlation between cc(A, P); cc(A, G) for non-informational groups with LGT and even a drop when only putative transfers from Archaea are considered. We noted also that, the non-informational groups without the above defined signature of LGT events show similar basic characteristics as the non-informational groups with such signature LGT.

**Table 2:** Correlation (R^2 ^value) between correlation coefficients for ortholog groups with putative LGA.

Coefficient of determination (R^2^) between:	informational groups	non-informational groups			All Groups with LGT from A
		all non-informational	putative LGT	remaining non-informational	

cc(A, P); cc(A, G)	0.54	0.68	0.67	0.77	0.57
cc(G, P); cc(G, A)	0.12	0.27	0.40	0.53	0.46
cc(P, G); cc(P, A)	0.02	0.31	0.44	0.23	0.43

### 2.5. In-silico Lateral Gene Transfers (s-LGT) elucidate unifying role of Lateral Gene Transfer

**Figure 4:**
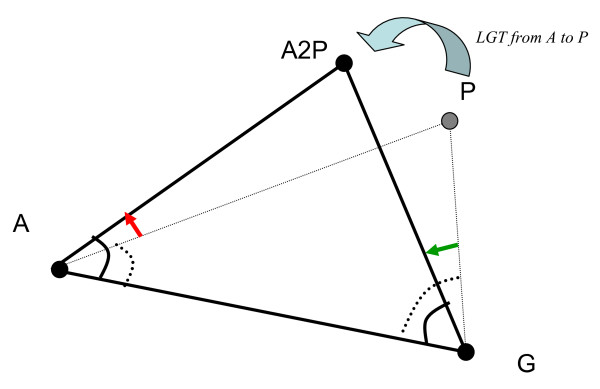
**Graphical illustration of the unifying role of s-LGT from A to P: *decreasing *the value of the R^2 ^for (*cc*(*A*, *P*),*cc*(*A*, *G*)) corresponds to *increasing *the angle A and X and increasing the value of the R^2 ^for (*cc*(*G*, *A*), *cc*(*G*, *P*)) corresponds to *decreasing *the angle G resulting in shifting the triangle towards being closer to equilateral.** The result of s-LGT from A to G is symmetrical.

We then explored more deeply this relation between LGT from Archaea to bacteria and the evolutionary pressure. Specifically, we performed a series of in-silico lateral gene transfers, s-LGT, where a random sequence from Proteobacteria or Gram-positive bacteria was replaced by a random sequence from Archaea. This process was repeated 100 times. Trends from the in-silico experiment agree with the trends seen in the real data (Table 3). LGT does not always increase the correlation between the values of *cc*(*N*, *X*) and *cc*(*N*, *Y*) but can been seen as a unifying force within ortholog group as illustrated in Figure 4. That is, if we think of the correlation between (*cc*(*N*, *X*), *cc*(*N*, *Y*)) as a measure of the angle between (N, X) and (N, Y) then s-LGTs from Archaea shifts the triangle A, P, G towards the equilateral shape (Figure 4).

**Table 3:** Results of in silico LGT (s-LGT) from Archaea to one of the bacterial clades (A2G or A2P). R^2 ^values for s-LGT are the average over 100 simulations.

correlation between:	R^2^	p-value	z-score
cc(A, G); cc(A, P)	0.783		
cc(A, A2G);cc(A, P)	0.719	0.02	1.86
cc(A;G);cc(A, A2P)	0.677	< 0.01	2.12
			
cc(P, G); cc(P, A)	0.187		
cc(P, A2G); cc(P, A)	0.403	< 0.01	3.94
			
cc(G, P); cc(G, A)	0.269		
cc(G, A2P); cc(G, A)	0.469	< 0.01	4.62

## 3. Conclusion

We considered the question if the evolutionary pressure within a set of ortholog groups is act uniformly in different subgroups of each ortholog group. We observed a distinctly different behavior of two subsets of our ortholog set, namely the informational and non-informational ortholog groups. We established, through a number of measurements, that evolutionary pressure acting on non-informational proteins is more uniform relative to their informational counterparts. The informational proteins show lower level of correlation between entropy profiles of the subgroups. In contrast, the non-informational groups display higher correlation between entropy profiles, and show more uniformity across subgroups. This uniformity of the evolutionary pressure acting on the non-informational groups might allow gene exchange through LGT. In contrast, the correlation between entropy profiles for informational groups is very low. This suggested that the informational proteins are not expected to be easily exchangeable between species. We confirmed that this is indeed the case for the ortholog groups under study. Thus the low level of lateral gene transfers for informational groups might be a result of distinct evolutionary pressure acting on these highly conserved groups.

It is important to bear in mind that the set of ortholog groups considered in this study is very biased – all groups are highly conserved with unique homolog in each genome used in the study. In particular the behavior of the non-informational groups considered in this study does not necessarily generalize across all proteins that excluded from the informational groups as defined here. The informational ortholog groups in our study do not cover all proteins related to information processing that are present in these organisms. A substantial fraction of these like specific transcription factors are left out because they do not satisfy the conservation criteria used in our study.

Jain et al. proposed that genes involved in multi-protein complexes are expected to be rarely transferred [14]. Indeed, the fact that many informational proteins are functioning as parts of multi-protein complexes is likely to contribute to the unique selective constraints acting on them. However a number of the ortholog groups in the non-informational set also form complexes with other proteins (e.g. NCAIR mutase, FGAM synthetase and glutamine amidotransferase domains, Molybdenum cofactor biosynthesis enzymes). Yet, the properties, such as correlation between entropy profiles, of these proteins are consistent with other proteins in the non-informational groups rather then with those in the informational groups. This indicates that the selective constraints in the informational groups have been imposed by a broader spectrum of conditions and not just being present in complexes.

Alternatively, the difference in the correlation in entropy profiles is a consequence of insufficient sampling of sequence space in the informational groups due reduced LGT, rather than a signature of a difference in evolutionary pressure. Such a possibility cannot be completely excluded. However, it less unlikely that LGT is the primary cause of those differences. For example, the difference in the *average entropy *of informational and non-informational sets is not statistically significant so the level of exploration of the sequence space is not drastically different. Furthermore, LGT is a stochastic process, with both informational and non-informational groups having a comparable chance of being transferred. It is at the level of the fixation of the transferred gene depending on its ability to function non-disruptively or to the advantage of the host system that differences could arise between the two classes. Numerous cases of horizontal transfer were reported amongst informational proteins, although less frequently than in the non-informational set [15-18]. Thus LGT between informational proteins is readily possible. The difference in frequency of LGT is consistent with the proposed differences in selective constraints which results in rejection of most such transfers in a certain subset of the informational genes.

These observations combined with our results suggest that the evolutionary pressure acting on the informational ortholog groups is not uniform across different sub-groups of organisms in this study. This might suggest "fine-tuning" of these informational group proteins in each lineage that makes them less exchangeable between lineages. In part this might relate to them functioning as parts of multi-protein complexes with several distinct subunits conserved subunits. In contrast, the non-informational groups might not experience such lineage-specific differences in selective pressure as they usually catalyze individual reactions in metabolic pathways with the flux of substrates mediating most functional interactions between them.

## 4. Methods

### Dataset

The set of ortholog groups from the COG database [12] was used. The COG database contains a total of 4873 clusters of orthologous groups (COGs) of proteins. Only COGs containing at least *n *representative organisms each in Archaea, Proteobacteria and Grampositive bacteria were considered. To ensure that the ortholog groups selected for this study contain sufficiently diverse organisms (to minimize the impact of redundancy during entropy calculations), we considered only those COGs containing organisms listed in Table 4. Because of this limitation, the number of COGs under consideration fell to 63 and 41 for *n *= 4 and 6, respectively (the complete list is given in Additional file 2).

**Table 4:** List of organisms used in the study; set4org contains genomes used in ortholog groups that span four organisms in each subgroup (A, G, or P) and set6org contains genomes used in ortholog groups that spans six organisms for each subgroup.

Group	Organism
Archaea	▶ Archaeoglobus fulgidus
	▶ Halobacterium sp. NRC-1
	▶ Methanococcus jannaschii
	▶ Pyrobaculum aerophilum
	Pyrococcus abyssi
	Thermoplasma acidophilum
Proteobacteria	▶ Caulobacter vibrioides
	▶ Escherichia coli O157:H7
	▶ Pseudomonas aeruginosa
	▶ Sinorhizobium meliloti
	Haemophilus influenzae
	Rickettsia prowazekii
Grampositive bacteria	▶ Bacillus subtilis
	▶ Escherichia coli O157:H7
	▶ Pseudomonas aeruginosa
	▶ Sinorhizobium meliloti
	Haemophilus influenzae
	Rickettsia prowazekii

▶ genomes included in *set4org*

### Constructing the multiple sequence alignments

**Figure 5:**
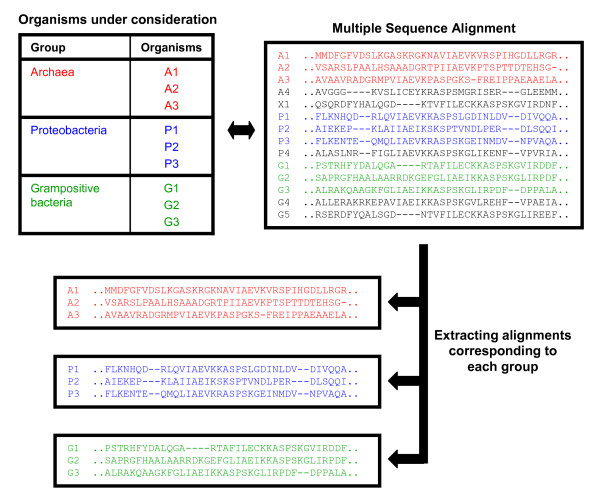
Flow chart for data generation.

Multiple sequence alignment (MSA) of the protein sequences in a COG were constructed using MUSCLE [19]. For each ortholog group, MSAs corresponding to only those organisms in Archaea, Proteobacteria, and Gram positive bacteria (Table 4) were constructed by extracting the corresponding set of rows in the ortholog group alignment (Figure 5).

### Constructing the entropy profiles and computing the correlation between amino-acid entropy profiles

The entropy of each column in an alignment is computed using AL2CO [20] with default parameters. Each alignment is represented using a conservation profile of length equal to the length of the alignment, with the *i*^th ^entry in the profile representing the entropy of residues in the *i*^th ^position of the alignment and is computed as $C(i)=\sum_{a=1}^{20}f_{a}(i)\ln f_{a}(i)$ where *f*_*a*_(*i*) is the frequency of amino acid a at position i. The entries are normalized by subtracting the mean and dividing by standard deviation. In order to eliminate the impact of gapped positions in the alignments, we only consider those residue positions (in the alignment) with < 50% gap in at least 2 of the 3 group alignments.

Each ortholog group under consideration is represented using a set of three entropy profiles corresponding to the three subgroups under consideration. The pairwise correlation of these 3 profiles is computed using Pearson's linear correlation coefficient.

### Computing the evolutionary distance

For each ortholog group, the distance matrix is computed from the multiple sequence alignment obtained with MUSCLE [19] using two approaches: (i) maximum likelihood method using quartet puzzling approach [21,22]. and (ii) the programs available in the ClustalW suite [23] (no correction for multiple substitutions was applied) where the distances are computed based on percent identity scores. Positions with gaps are excluded as described as described in computing entropy profile subsection. The computed distances ware highly correlated (r^2 ^between 0.95 and 0.98). Results included in the paper are obtained using maximum likelihood method.

**Figure 6:**
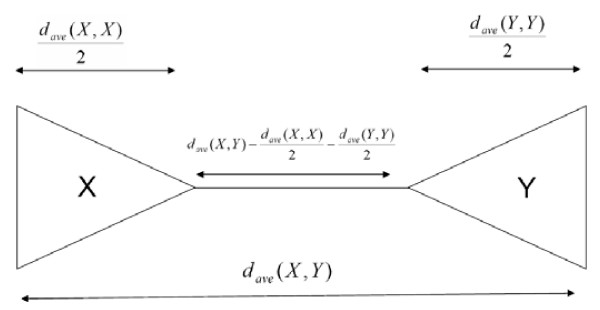
Computation of *r*(*X*, *Y*) as the ratio of the approximation of the distances between the roots of subtree spanned by *X *and subtree spanned by *Y*, $d_{ave}(X,Y)-\frac{1}{2}d_{ave}(X,X)-\frac{1}{2}d_{ave}(Y,Y)$, the average distance *d*_*ave*_(*X*, *Y*) of the sequences in the two subgroups.

The average evolutionary distance between a member in subgroup *X *and a member in subgroup *Y *is given by

$$d_{ave}(X,Y)=\frac{\sum_{i\in X}\sum_{j\in Y}dist(i,j)}{f(X,Y)},$$

where *f*(*X*, *Y*) = |*X*||*Y*| if *X *≠ *Y*, and *f*(*X*, *Y*) = |*X*|! if *X *= *Y*. Recall that |*X*|, |*Y*| = *n*. The relative group distance *r*(*X*, *Y*), measuring the distance between the ancestors of members in subgroup X and Y with respect to *d*_*ave*_(*X*, *Y*), is given by

$$r(X,Y)=\frac{d_{ave}(X,Y)-\frac{d_{ave}(X,X)}{2}-\frac{d_{ave}(Y,Y)}{2}}{d_{ave}(X,Y)},$$

where the numerator approximates the distance between the ancestors of members in subgroup *X *and members in subgroup *Y *(see Figure 6).

### Lateral Gene Transfers

To identify putative LGT events, evolutionary tree for (12-species) ortholog groups were constructed using ClustalW suite [23], displayed with the TreeView program [24] and checked manually for disagreements with the species tree. Putative transfer from Archaea was assigned if a single bacterial gene was found in the Archaeal clade.

## Authors' contributions

TMP participated in designing the study, analysis and interpretation of the data, performed a part of the computations, and drafted the manuscript. RJ performed most of the computations and participated in data analysis. LA participated in data analysis, interpreted the data, and participated in writing the paper. DJL conceived the study, participated in analysis and interpretation the data. All authors read and approved the final manuscript.

## Supplementary Material

Additional file 1 The dependence between correlation coefficient computed for the four element subgroups and six element subgroups for pairs (A, P) and (PG). The data provided shows graphs of dependences between correlation coefficient computed for the four element subgroups and six element subgroups for pairs (A, P) and (PG) Click here for file

Additional file 2 List of COGs. The table of COGs used in the experiment. Those listed in bold are used to show the correlation between 4- and 6- species groups Click here for file
