# Supplementary material for: Differences in evolutionary pressure acting within highly conserved ortholog groups
Source: BMC Evol Biol. 2008 Jul 17;8:208. doi: 10.1186/1471-2148-8-208 (PMC2488352; doi:10.1186/1471-2148-8-208)
Supplement: Additional file 1 — The dependence between correlation coefficient computed for the four element subgroups and six element subgroups for pairs (A, P) and (PG). The data provided shows graphs of dependences between correlation coefficient computed for the four element subgroups and six element subgroups for pairs (A, P) and (PG) [file 1471-2148-8-208-S1.pdf]

The dependence between correlation coefficient computed for the four element subgroups and six element subgroups

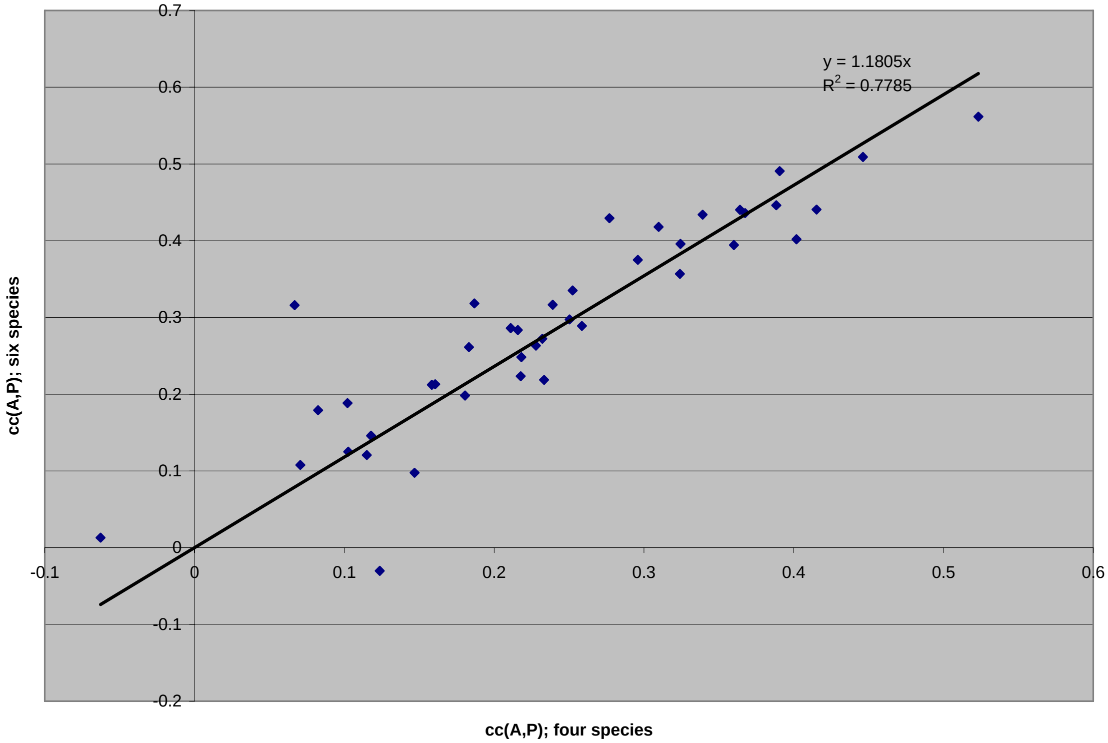

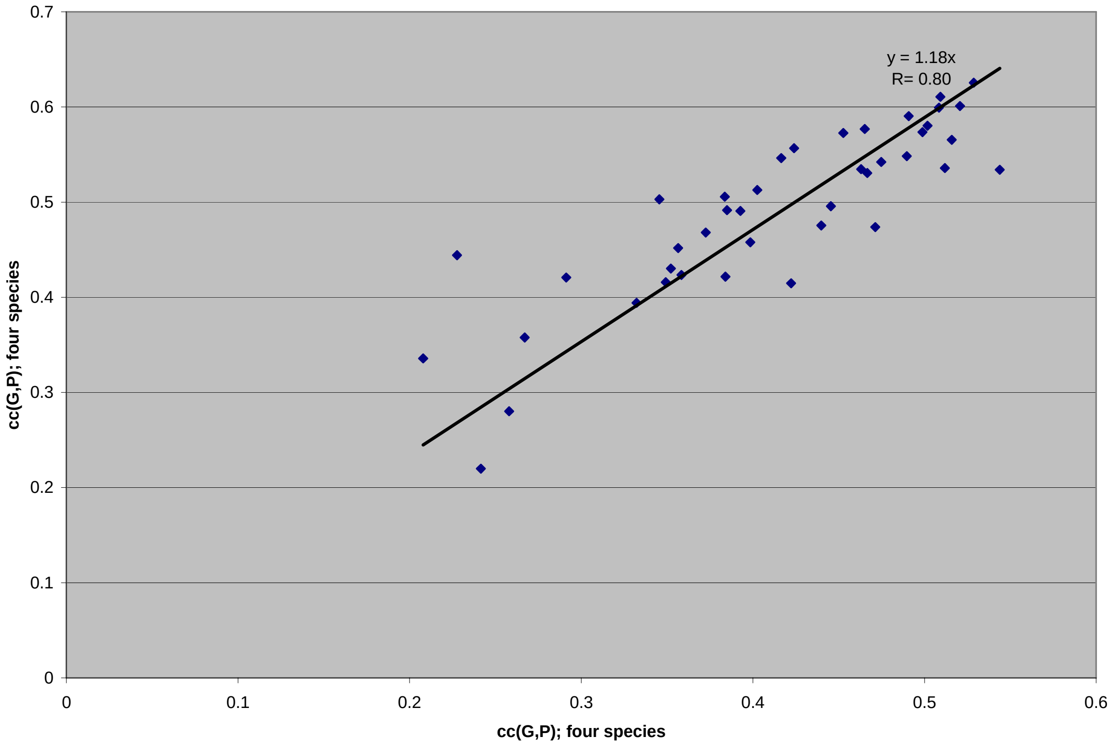

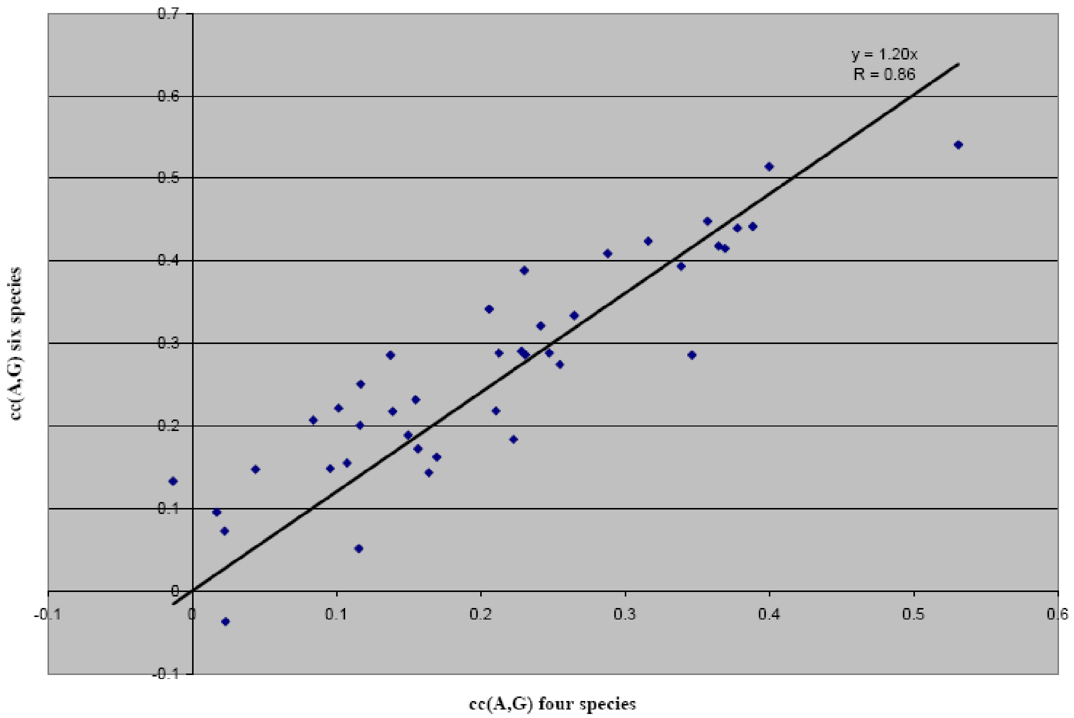

y = 1.20x
R = 0.86
cc(A,G) six species
cc(A,G) four species
0.7
0.6
0.5
0.4
0.3
0.2
0.1
0
-0.1
0.1
0.2
0.3
0.4
0.5
0.6
