# Supplementary material for: Differences in evolutionary pressure acting within highly conserved ortholog groups
Source: BMC Evol Biol. 2008 Jul 17;8:208. doi: 10.1186/1471-2148-8-208 (PMC2488352; doi:10.1186/1471-2148-8-208)
Supplement: Additional file 2 — List of COGs. The table of COGs used in the experiment. Those listed in bold are used to show the correlation between 4- and 6- species groups [file 1471-2148-8-208-S2.pdf]

List of COGs

Those listed in bold are used to show the correlation between 4- and 6- species groups

**COG0013**	Alanyl-tRNA synthetase
**COG0016**	Phenylalanyl-tRNA synthetase alpha subunit
COG0041	Phosphoribosylcarboxyaminoimidazole (NCAIR) mutase
COG0046	Phosphoribosylformylglycinamidine (FGAM) synthase, synthetase domain
COG0047	Phosphoribosylformylglycinamidine (FGAM) synthase, glutamine amidotransferase domain
**COG0048**	Ribosomal protein S12
**COG0049**	Ribosomal protein S7
**COG0052**	Ribosomal protein S2
**COG0060**	Isoleucyl-tRNA synthetase
**COG0072**	Phenylalanyl-tRNA synthetase beta subunit
COG0077	Prephenate dehydratase
**COG0081**	Ribosomal protein L1
COG0082	Chorismate synthase
**COG0087**	Ribosomal protein L3
**COG0088**	Ribosomal protein L4
**COG0089**	Ribosomal protein L23
**COG0090**	Ribosomal protein L2
**COG0091**	Ribosomal protein L22
**COG0092**	Ribosomal protein S3
**COG0093**	Ribosomal protein L14
**COG0094**	Ribosomal protein L5
**COG0096**	Ribosomal protein S8
**COG0097**	Ribosomal protein L6P/L9E
**COG0098**	Ribosomal protein S5
**COG0099**	Ribosomal protein S13
**COG0101**	Pseudouridylate synthase
**COG0102**	Ribosomal protein L13
**COG0103**	Ribosomal protein S9
COG0105	Nucleoside diphosphate kinase
COG0106	Phosphoribosylformimino-5-aminoimidazole carboxamide ribonucleotide (ProFAR) isomerase
COG0113	Delta-aminolevulinic acid dehydratase
**COG0124**	Histidyl-tRNA synthetase
COG0131	Imidazoleglycerol-phosphate dehydratase
COG0134	Indole-3-glycerol phosphate synthase
COG0135	Phosphoribosylanthranilate isomerase
COG0137	Argininosuccinate synthase
COG0139	Phosphoribosyl-AMP cyclohydrolase
COG0150	Phosphoribosylaminoimidazole (AIR) synthetase
COG0151	Phosphoribosylamine-glycine ligase
COG0159	Tryptophan synthase alpha chain
**COG0164**	Ribonuclease HII
COG0181	Porphobilinogen deaminase
**COG0185**	Ribosomal protein S19
**COG0197**	Ribosomal protein L16/L10E
**COG0198**	Ribosomal protein L24

**COG0200**	Ribosomal protein L15
**COG0202**	DNA-directed RNA polymerase, alpha subunit/40 kD subunit
**COG0244**	Ribosomal protein L10
**COG0255**	Ribosomal protein L29
**COG0256**	Ribosomal protein L18
COG0284	Orotidine-5'-phosphate decarboxylase
COG0287	Prephenate dehydrogenase
COG0315	Molybdenum cofactor biosynthesis enzyme
**COG0442**	Prolyl-tRNA synthetase
COG0519	GMP synthase, PP-ATPase domain/subunit
**COG0525**	Valyl-tRNA synthetase
**COG0528**	Uridylate kinase
**COG0533**	Metal-dependent proteases with possible chaperone activity
COG0540	Aspartate carbamoyltransferase, catalytic chain
**COG0541**	Signal recognition particle GTPase
**COG0552**	Signal recognition particle GTPase
**COG1758**	DNA-directed RNA polymerase, subunit K/omega
**COG1841**	Ribosomal protein L30/L7E
